# Genomic organization and recombination analysis of a porcine sapovirus identified from a piglet with diarrhea in China

**DOI:** 10.1186/s12985-017-0729-1

**Published:** 2017-03-16

**Authors:** Jingjiao Li, Quan Shen, Wen Zhang, Tingting Zhao, Yi Li, Jing Jiang, Xiangqian Yu, Zhibo Guo, Li Cui, Xiuguo Hua

**Affiliations:** 1 School of Agriculture and Biology, Shanghai Jiao Tong University, Shanghai, 200240 China; 2 School of Medicine, Jiangsu University, Zhenjiang, 212013 China; 3 Shanghai Entry-Exit Inspection and Quarantine Bureau, Shanghai, 200135 China; 4 Shanghai Pudong New Area Center for Animal Disease Control and Prevention, Shanghai, 200136 China; 5 Shanghai Pudong New Area Center for Agriculture Service, Shanghai, 201202 China

**Keywords:** Porcine sapovirus, Genome organization, Recombination

## Abstract

**Background:**

Sapovirus (SaV), a member of the family *Caliciviridae*, is an etiologic agent of gastroenteritis in humans and pigs. To date, both intra- and inter-genogroup recombinant strains have been reported in many countries except for China. Here, we report an intra-genogroup recombination of porcine SaV identified from a piglet with diarrhea of China.

**Methods:**

A fecal sample from a 15-day-old piglet with diarrhea was collected from Shanghai, China. Common agents of gastroenteritis including porcine circovirus type 2, porcine rotavirus, porcine transmissible gastroenteritis virus, porcine SaV, porcine norovirus, and porcine epidemic diarrhea virus were detected by RT-PCR or PCR method. The complete genome of porcine SaV was then determined by RT-PCR method.

Phylogenetic analyses based on the structural region and nonstructural (NS) region were carried out to group this SaV strain, and it was divided into different genotypes based on these two regions. Recombination analysis based on the genomic sequence was further performed to confirm this recombinant event and locate the breakpoint.

**Results:**

All of the agents showed negative results except for SaV. Analysis of the complete genome sequence showed that this strain was 7387 nt long with two ORFs and belonged to SaV GIII. Phylogenetic analyses of the structural region (complete VP1 nucleotide sequences) grouped this strain into GIII-3, whereas of the nonstructural region (RdRp nucleotide sequences) grouped this strain into GIII-2. Recombination analysis based on the genomic sequence confirmed this recombinant event and identified two parental strains that were JJ259 (KT922089, GIII-2) and CH430 (KF204570, GIII-3). The breakpoint located at position 5139 nt of the genome (RdRp-capsid junction region). Etiologic analysis showed the fecal sample was negative with the common agents of gastroenteritis, except for porcine SaV, which suggested that this recombinant strain might lead to this piglet diarrhea.

**Conclusions:**

P2 strain was an intra-genogroup recombinant porcine SaV. To the best of our knowledge, this study would be the first report that intra-genogroup recombination of porcine SaV infection was identified in pig herd in China.

## Background

Sapovirus (SaV) is the causative agent of gastroenteritis and has been detected in multiple mammalian species and pigs are the predominant host of SaV [1–3]. Based on the complete capsid protein VP1 sequences, SaV now has been divided into 15 genogroups (GI-GXV) and GIII was the predominant one infecting pigs [4]. GIII strains have been further clustered into several genotypes based on the partial VP1 or RNA-dependent RNA polymerase (RdRp) sequences reported by different researchers [5, 6]. Genomic organization of a common SaV includes two open reading frames (ORF1 and ORF2), whereas in certain genogroups strains were identified an additional ORF (ORF3) [7–9]. ORF1 encodes the predicted viral NS proteins and the major capsid protein VP1, ORF2 encodes the minor capsid protein VP2, and ORF 3 encodes a small basic protein with unknown function [9].

SaV strains with inconsistent grouping between the nonstructural protein-encoding region (including the RdRp region) and the VP1 encoding region have been designated as “recombinant”. Previous studies suggested that the recombination site was at the polymerase-capsid junction [1, 2]. To date, both intra- and inter-genogroup recombinant strains have been reported in humans, however, few porcine recombinant SaV strains have been reported all over the world [10–13]. In particular, no recombinant strain of SaV has been identified either in human or pig in China.

In the present study, we characterized the complete genome of a porcine SaV which might lead to a piglet diarrhea and analyzed the recombination of this strain. Phylogenetic and recombination analysis showed that this strain was an intra-genogroup recombinant, and the breakpoint for this recombination event located at the polymerase-capsid junction within ORF1. This is the first report that intra-genogroup recombination of porcine SaV related with a piglet diarrhea in China.

## Methods

### Specimens

A fecal sample from a 15-day-old piglet with diarrhea was collected in October, 2015 in Shanghai, China. Bacterial infection was ruled out by the diagnosis of the licensed veterinarian from the pig farm. In order to avoid sample contamination, specimen was obtained directly from the pig anus and disposable materials were used during sampling. Stool sample was freshly collected and immediately converted to 10% (w/v) suspension in phosphate-buffered saline (PBS, 0.01 M, pH 7.2-7.4) for further RNA and DNA extraction.

### RNA and DNA extraction

Viral RNA/DNA was extracted from 200 μl of fecal supernatant by using the TaKaRa MinBEST Viral RNA/DNA Extraction Kit version 5.0 (TaKaRa, Japan), according to the manual instruction. RNA/DNA was dissolved in 25 μl RNase-free water and reverse transcription was performed immediately.

### RT-PCR or PCR

Polymerase chain reaction (PCR) or reverse transcription polymerase chain reaction (RT-PCR) assays with certain primer sets for the detection of porcine common viruses that may cause pig diarrhea including porcine circovirus type 2 (PCV2), porcine rotavirus (PRV), porcine transmissible gastroenteritis virus (PTGV), porcine SaV, porcine norovirus (NoV), and porcine epidemic diarrhea virus (PEDV) were performed as previously described [14–16].

### Whole genome amplification

To amplify the genome of this SaV strain, the first strand cDNA was synthetized in 20 μl reaction mixture followed the Thermo Scientific RevertAid First Strand cDNA Synthesis Kit’s (Thermo, USA) introductions with 1 μl random hexamer primer supplied by the kit for the full sequences amplification or 1 μl gene-specific primer QT (Table 1) especially for the 3’ end amplification (3’ RACE method), respectively [17]. Eleven sets of specific primers were designed based on KF204570 to amplify the remaining sequences (Table 1). All the PCR products were purified by OMEGA Gel Kit (OMEGA, USA) following the manufacturer’s instructions, ligated to the pMD19-T vector (TaKaRa, Japan) and then transformed into DH5α competent *Escherichia coli* cells (Yeasen, China). For each product, three to five colonies were selected and sequenced (Sangon, China) in both directions with the M13+/- universal primers. The consensus sequences were assembled using the Lasergene Software package (version 8) (DNASTAR Inc., Madison, WI).

**Table 1:** Primers for amplifying the complete genome

Primer name	Primer sequence (5’-3’)	Position	References
SAVP1F	GTGATCGTGATGGCTAATTGC	1-21	JX678943
SAVP1R	GAACTGTTTCAACACTGT	622-639	JX678943
SAVP2F	GGGACATGTGGCAGTAC	533-549	JX678943
SAVP2R	TTGAAGTAGTCCACTATCCACAT	1087-1109	JX678943
SAVP3F	ACTGACAAGTTTGCTGA	862-878	JX678943
SAVP3R	GTGTGGGGCAATTGGTGGT	1660-1678	JX678943
SAV4FW	TTGAATTGTGACCGGCCAGAG	1600-1620	KX688107
SAVP5R	TCATCATACTCATCGTCCCT	2863-2882	JX678943
SAVP6F	CTGAACACCCGTGA	2782-2795	JX678943
SAVP9R	AGCACAGCCATGGCAAAG	4551-4568	JX678943
SAVP10F	CCATAGCCACACTGTGTTCAC	4427-4447	JX678943
SAVP10R	TCTTCATCTTCATTGGTGGGAG	5102-5123	JX678943
SAVP11F	CCAAGGGCAGTGTTTGAC	4963-4980	JX678943
SAVP11R	GGTTGGTACACATAAAGTGCC	5676-5696	JX678943
SAVP12F	GATGTTAGGGCGGTGGA	5620-5636	JX678943
SAVP12R	AGGTGAAAGTGGTGTCTTCTG	6674-6694	JX678943
SAVP13F	CTCGGCACGCACACGGG	6469-6485	JX678943
SAVP13R	TGATTGGCAGGTAAATTTG	6961-6979	JX678943
SAV14N	GATGGAGTTGGCTAAAGAACA	6893-6913	KX688107
Q_T_	CCAGTGAGCAGAGTGACGAGGACTCGAGCTCAAGCTTTTTTTTTTTTTTTTT		[17]
Q_O_	CCAGTGAGCAGAGTGACG		[17]
Q_I_	GAGGACTCGAGCTCAAGC		[17]

### Sequence and recombination analysis

Similarity searches of the sequences were carried out in BLAST (http://www.ncbi.nlm.nih.gov/BLAST/). After a multiple alignment with CLUSTAL W embedded in MEGA 7, the phylogenetic relationship of the strain in the present study and the reference sequences were assessed using MEGA 7. For analysis in MEGA 7, Jukes-cantor (JC) distance was utilized, employing the Neighbor joining (NJ) algorithm [18]. The reliability of different phylogenetic groupings was evaluated by using the bootstrap test (1000 bootstrap replications) available in MEGA 7. The identification of recombinants was performed by using the Recombination Detection Program 4 (RDP 4) (http://darwin.uvigo.es/rdp/rdp.html) [19]. Prototype SaV strains used as references in the analysis with their corresponding GenBank accession numbers, source of origin and genogroups are showed in Table 2.

**Table 2:** Profile of porcine sapovirus isolates used for sequence analyses

Strain ID	GenBank accession number	Length	Geographic origin	Genogroup
VZ	DQ056363	1940 bp	Venezuela	GIII
YiY1/2006	EU381231	1635 bp	**China** ^b^	GIII
OH-MM280/2003	AY823308	2971 bp	USA	GIII
NC-QW270	AY826426	2971 bp	USA	GIII
S20	AB242875	1635 bp	Japan	GIII
HW20/2007	HM346629	2983 bp	South Korea	GIII
DG24/2007	HM346628	3016 bp	South Korea	GIII
ah-1/2009	JX678943	7342 bp	**China** ^b^	GIII
Cowden	AF182760	7320 bp	USA	GIII
LL14	AY425671	7291 bp	USA	GIII
JJ259	KT922089	7347 bp	USA	GIII
sav1/2008	FJ387164	7558 bp	**China** ^b^	GIII
CH430/2012	KF204570	7371 bp	**China** ^b^	GIII
p2^a^	KX688107	7371 bp	**China** ^b^	GIII
WG194D-1	KX000383	7496 bp	USA	GV
TYMPo239	AB521771	3949 bp	Japan	GV
TYMPo31	AB521772	3949 bp	Japan	GV
OH-JJ674	KJ508818	7198 bp	USA	GVI
OH-JJ681	AY974192	7198 bp	USA	GVI
RV0042/2011	KX000384	7150 bp	USA	GVII
OH-LL26/2002	AY974195	2952 bp	USA	GVII
K7	AB221130	7144 bp	Japan	GVII
K10	AB221131	2972 bp	Japan	GVII
AB23	FJ498787	3000 bp	Canada	GVII
DO19/2007	HM346630	2983 bp	South Korea	GVII
2014P2	DQ359099	1626 bp	Brazil	GVII
F2-4/2006	GU230161	2935 bp	Canada	GVII
F8-9/2006	GU230162	2926 bp	Canada	GVII
WGP3/2009	KC309420	2933 bp	USA	GVII
WGP247/2009	KC309421	6052 bp	USA	GVII
WG194D/2009	KC309416	6654 bp	USA	GVIII
WG214D/2009	KC309419	7497 bp	USA	GVIII
06-18p3	EU221477	3094 bp	Italy	GVIII
F19-10	FJ498786	3142 bp	Canda	GVIII
WG180B	KC309415	3111 bp	USA	GVIII
WG197C/2009	KC309417	6497 bp	USA	GVIII
WG214C	KC309418	3695 bp	USA	GIX
F16-7	FJ498788	2982 bp	Canada	GIX
K8	AB242873	1617 bp	Japan	GX
2053P4	DQ359100	1635 bp	Brazil	GXI

^a^The strain with complete genomic sequence determined in this study

^b^The strains which were deposited in GenBank database by Chinese researchers and used in this study were bolded

## Results

### Result of viruses detection

RT-PCR or PCR were performed to detect the common viruses that may cause pig diarrhea. Result showed that the fecal sample was negative for porcine NoV, PCV 2, PRV, PTGV and PEDV, but was positive for porcine SaV.

### Genome organization

**Fig. 1:**
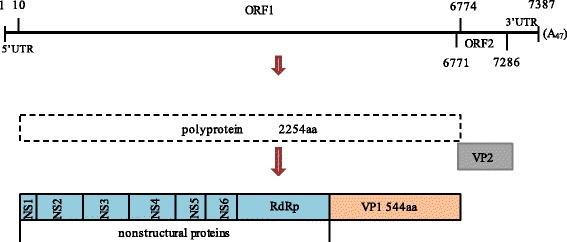
Schematic of strain p2 genome organization. The full genome of strain p2 is a linear, positive-sense,single-stranded RNA of 7387 bp with a poly (A) tail at the 3’ end. The nucleotides coordinate of 5’ UTR, ORF 1 and ORF 2, and 3’ UTR are indicated on the map. ORF1 encoded a single polyprotein which was subsequently cleaved into seven nonstructural proteins containing the RdRp region and the major capsid protein VP1. ORF2 encoded the minor capsid protein VP2 (171 aa)

The whole genome of p2 strain was determined by RT-PCR and 3’ RACE method. The entire genome of this porcine SaV strain (named as p2, GenBank no. KX688107) consisted of 7387 nucleotides (nt) including the poly (A) tail with a 9 nt 5’ untranslated region (UTR) and a 54 nt 3’-UTR. Similar to previously reported porcine strains, p2 has two ORFs. ORF1 comprised 6,765 nt (10-6774) encoding a single polyprotein of 2,254 amino acid (aa). ORF2 comprised 516 nt (6771–7286) and contained a 4-nt overlapping region (^6771^ATGA^6774^) with the 3′ end of ORF1 (Fig. 1).

### Phylogenetic and recombination analysis

P2 shared the highest nucleotide homology (91%) through the entire genome and about 94% in the capsid region with CH430 (GIII-3, GenBank no. KF204570) (a Chinese porcine SaV strain), respectively. However, it shared only 87% identity with strain CH430 in the RdRp region. On the contrary, p2 shared the highest 91% nucleotide identity with an American strain JJ259 (GIII-2, GenBank no. KT922089) in the RdRp region, which was opposite to the general phenomenon observed for caliciviruses that the RdRp region is more conserved than the capsid region. In the previous studies, SaV strains with inconsistent grouping between the nonstructural protein-encoding region (including the RdRp region) and the VP1 encoding region were designated as recombinant. All of these suggested that p2 strain may be a recombinant virus.

**Fig. 2:**
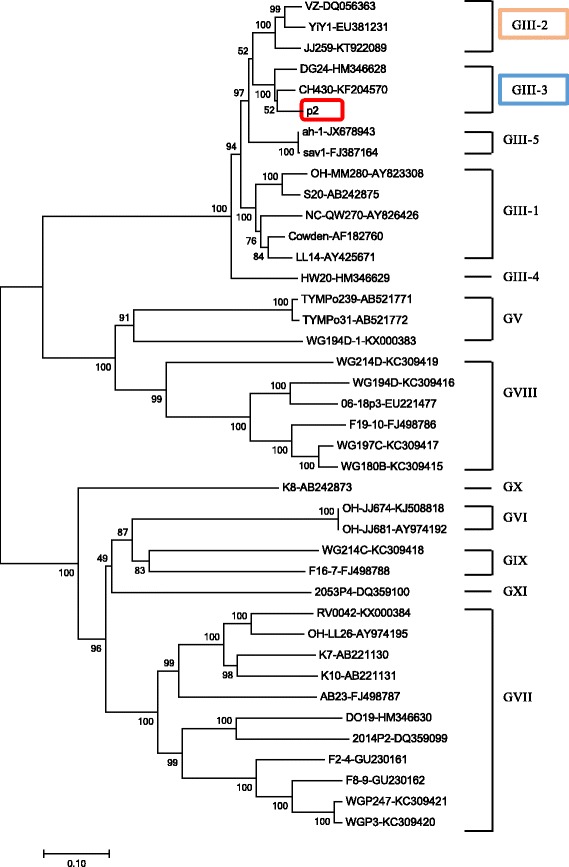
Phylogenetic tree based on the complete VP1 nucleotide sequences of 42 SaV strains using MEGA 7. These strains represented all reported 8 genogroups (GIII, GV-GXI) of porcine SaV. GIII strains were grouped into 5 genotypes (GIII-1 – GIII-5). Among those strains, the p2 strain determined in this study was marked with box. Each SaV strain is presented as the following format: strain ID- GenBank accession number

**Fig. 3:**
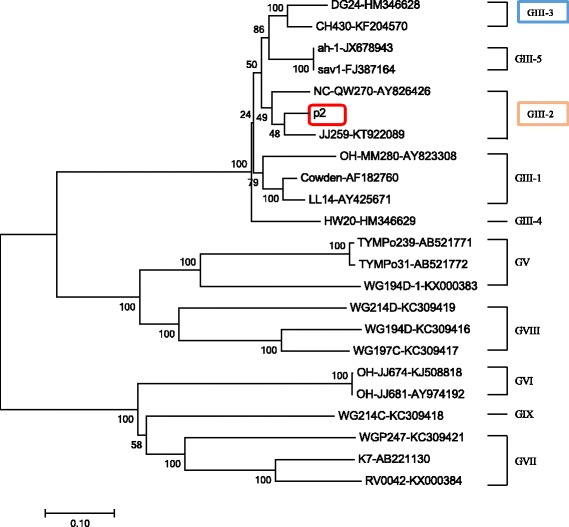
Phylogenetic tree of the 3’ end of RdRp nucleotide sequences (780 bp) of SaV strains. Only 25 of 42 strains had the available sequences of 3’ end of the RdRp region covering the RdRp-capsid junction and GIII strains were grouped into 5 genotypes (GIII-1-GIII-5). The strain p2 reported in this study was indicated in box

**Fig. 4:**
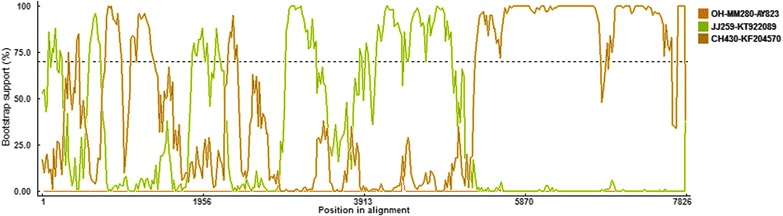
Identification of recombinant p2 strain based on RDP 4. BOOTSCAN evidence for the recombination origin on the basis of pairwise distance, modeled with a window size 200, step size 25, and 100 Bootstrap replicates

Phylogenetic and recombination analysis were further performed to verify the genotype definition and recombination. Phylogenetic tree based on 42 of the complete VP1 nucleotide sequences was constructed by the NJ method, in which p2 was grouped into GIII-3 clustering with CH430 (Fig. 2). However, phylogenetic analysis based on the 3’ end of RdRp nucleotide sequences gave a different grouping result, in which p2 was grouped into the GIII-2 clustering with JJ259 (Fig. 3). This finding suggested that this strain may be an intra-genogroup recombinant within GIII. To confirm the finding and detect the breakpoint where the recombination event occurred, we performed recombination analysis with p2 as the query sequence, JJ259 and CH430 as the background sequences and OH-MM280 (GIII-1, GenBank no. AY823308) as the outlier sequence using RPD software. Recombination analysis confirmed that the p2 strain was a recombinant and the major and minor parent was JJ259 and CH430, respectively. Moreover, the breakpoint for this recombination event located at position 5139 nt of the genome, which was the polymerase-capsid junction within ORF1 (Fig. 4).

## Discussion

SaV causes acute gastroenteritis in humans and animals including pigs, mink, dogs, sea lions, and bats [4, 9, 20]. Porcine SaV and human strains were separated into a different genogroups, however, based on analyses of RdRp or capsid protein genes, porcine SaV that genetically resembled human strains rather than previously recognized porcine strains had been identified [21, 22]. These findings suggested the possibility of a pig reservoir for human strains or vice versa. Meanwhile, recombination as an important survival event for all living creatures, may result in generation of new viruses with unknown pathogenic potential and altered species tropism for both animals and humans [23]. To date, both intra- and inter-genogroup recombinant strains have been reported [11–13, 22–24]. So far the recombinant strain was not found in either human beings or animals of China, although the SaV infections in children and pigs were common in this area [25–27]. Here, we reported a complete genome of a recombinant SaV strain that identified from a piglet with diarrhea in China. Phylogenetic and recombination analysis based on the genomic sequences showed that p2 was an intra-genogroup recombinant within GIII, and the breakpoint located in the RdRp-capsid junction region, which was consistent with most of other SaV recombination events. Previous reports had shown that, in the genome of SaV, recombination mostly occurred at the polymerase-capsid junction region within ORF 1 which was referred as ‘hot spot’ [2, 13]. Chang et al. detected a 2.2 kb RNA in vitro replication assay with the replication complex of SaV Cowden strain extracted from virus-infected cells suggesting that SaV will generate subgenomic RNA during virus replication [8]. Moreover, researchers found that the RdRp-capsid junction region of SaV contained a highly conserved ~20 nucleotide (nt) motif in both genomic and subgenomic RNA molecules which was considered as a transcription start signal [2]. This conserved nucleotide motif may facilitate homologous recombination during co-infection of a cell by different genogroups of virus.

SaV has been detected in a wide range of mammals with potential ability of zoonotic transmission [28–31]. Recombination was considered as a force of evolution which may produce new virus with potentially different pathogenesis and virulence [32]. Moreover, an infectious NoV chimera comprising the distinct biological properties from the parental viruses has been constructed and is infectious in vivo [32, 33]. Therefore, detection and understanding the recombination of SaV is important. In the current study, p2 strain was identified from a pig with diarrhea, in which bacteria and the common enteric viruses that cause pig gastroenteritis were ruled out. This suggested that this recombinant virus may cause this piglet diarrhea under the nature condition. More researches such as experimental infection should be performed in the future to confirm the pathogenicity of this recombinant strain.

## Conclusions

This is the first report that an intra-genogroup recombinant porcine SaV infected piglet in China and may lead to the piglet diarrhea. This finding raised our awareness of whether recombination in SaV will increase its virulence.

## Abbreviations

aa: Amino acid
GIII: Genogroup III
JC: Jukes-cantor
NJ: Neighbor joining
NoV: Norovirus
nt: Nucleotides
ORF: Open reading frame
PCR: Polymerase chain reaction
PCV2: Porcine circovirus type 2
PEDV: Porcine epidemic diarrhea virus
PRV: Porcine rotavirus
PTGV: Porcine transmissible gastroenteritis virus
RdRp: RNA-dependent RNA polymerase
RT-PCR: Reverse transcription polymerase chain reaction
SaV: Sapovirus
UTR: Untranslated region.
